# Primary care practice-based care management for chronically ill patients (PraCMan) in German healthcare: Outcome of a propensity-score matched cohort study

**DOI:** 10.1080/13814788.2021.1962280

**Published:** 2021-08-11

**Authors:** Jonas D. Senft, Tobias Freund, Michel Wensing, Simon Schwill, Regina Poss-Doering, Joachim Szecsenyi, Gunter Laux

**Affiliations:** Department of General Practice and Health Services Research, University Hospital Heidelberg, Heidelberg, Germany

**Keywords:** General practice, health care organisation and management

## Abstract

**Background:**

Growing prevalence of chronic diseases is a rising challenge for healthcare systems. The Primary Care Practice-Based Care Management (PraCMan) programme is a comprehensive disease management intervention in primary care in Germany aiming to improve medical care and to reduce potentially avoidable hospitalisations for chronically ill patients.

**Objectives:**

This study aimed to assess the effect of PraCMan on hospitalisation rate and related costs.

**Methods:**

A retrospective propensity-score matched cohort study was performed. Reimbursement data related to patients treated in general practices between 1st July 2013 and 31st December 2017 were supplied by a statutory health insurance company (AOK Baden-Wuerttemberg, Germany) to compare hospitalisation rate and direct healthcare costs between patients participating in the PraCMan intervention and propensity-score matched controls following usual care. Outcomes were determined for the one-year-periods before and 12 months after beginning of participation in the intervention.

**Results:**

In total, 6148 patients participated in the PraCMan intervention during the observation period and were compared to a propensity-score matched control group of 6148 patients from a pool of 63,446 eligible patients. In the one-year period after the intervention, the per-patient hospitalisation rate was 8.3% lower in the intervention group compared to control (*p* = 0.0004). Per-patient hospitalisation costs were 9.4% lower in favour of the intervention group (*p* = 0.0002).

**Conclusion:**

This study showed that the PraCMan intervention may be associated with a lower rate of hospital admissions and hospitalisation costs than usual care. Further studies may assess long-term effects of PraCMan and its efficacy in preventing known complications of chronic diseases.

KEY MESSAGES

- Primary Care Practice-Based Care Management (PraCMan) is a disease management intervention for chronically ill patients aiming to avoid unnecessary hospitalisations.
- A retrospective propensity-matched cohort study was performed to evaluate its efficacy.
- Participation in PraCMan may be associated with a lower of hospital admission rate and costs than usual care.

## Introduction

Growing prevalence of chronic diseases is a major challenge for healthcare systems. Physicians face increasing numbers of patients with complex healthcare needs while average consultation time in primary care is limited to less than 10 min in many countries [1]. This may eventually affect the quality of medical care of chronic conditions since improper management of, e.g. asthma exacerbations or wound healing disorders in diabetic patients may lead to adverse outcomes, potentially avoidable hospitalisation and increased healthcare costs [2].

In recent decades, various strategies have been developed to maintain access to high-quality primary care. Non-physician health workers like nurse practitioners in the US and Australia, licenced nurses in Denmark and The Netherlands, and certified healthcare assistants in Germany (‘Versorgungsassistent/in der Hausarztpraxis,’ VERAH) have been increasingly involved in patient care [3–6]. Nurse-led programmes have been developed to treat common consultations like gout flares or osteoarthritis [7,8] and complex disease management interventions have been developed to treat chronic health conditions [9–11].

The Primary Care Practice-Based Care Management (PraCMan) programme is a large-scale disease management intervention for chronically ill patients, which was introduced in Germany to improve medical care and reduce avoidable hospitalisations in the primary care setting [12]. Patients with chronic heart failure (CHF), type 2 diabetes (T2DM) or chronic obstructive pulmonary disease (COPD) who are at high risk for hospitalisation, can participate in this scripted intervention provided by specifically trained medical assistants under general practitioners’ (GP) guidance. Soon after establishment, the efficacy of PraCMan was analysed in an RCT including 115 general practices and 2076 patients [13]. No effect was shown on all-cause hospitalisations at 24 months. Nevertheless, quality of life and general health improved, indicating that the intervention may have a beneficial health effect.

To date, there is only limited knowledge about the efficacy of comprehensive interventions addressing multiple chronic index conditions. Subsequent to the PraCMan trial, the intervention was introduced into routine primary care in the federal state of Baden-Württemberg. High volume secondary data were analysed in this cohort study to reassess potential effects of the PraCMan intervention on the need for hospitalisation and its direct healthcare costs in the routine primary care setting.

## Methods

### Study design

A retrospective propensity-matched cohort study was performed. Data related to patients treated in general practices between 1st July 2013 and 31st December 2017 were supplied by a statutory health insurer (AOK, Baden-Wuerttemberg, Germany) to compare hospitalisation rate and costs between patients participating in the PraCMan intervention and matched patients following usual care.

### Ethics

Ethical approval was given by the local institutional Ethics Committee of the University Hospital Heidelberg (No. S-359/2013).

### Study population

The AOK is the largest statutory health insurer of the federal state of Baden-Wuerttemberg, covering nearly 4 million inhabitants. Secondary data related to patients participating in a GP-centred care programme (German: ‘Hausarztzentrierte Versorgung’ (HZV)) were eligible for data analysis. The HZV programme is a large-scale, legally stipulated health plan aiming to strengthen outpatient care by encouraging patients to enrol with a GP [14]. Secondary patient data was included in this analysis according to the following criteria: age 18 years or older, at least one visit to the primary care physician in the relevant year, continuous registration to HZV program. Patients were excluded if they were also registered in other primary care contracts (e.g. integrated care contracts). Patients participating in PraCMan (cases) were compared with patients not participating in PraCMan (controls).

### Intervention

PraCMan is a collaborative case management intervention aiming to strengthen outpatient care for chronically ill patients and reduce avoidable hospitalisations [12,13,15]. Practices participating in the HZV programme can enrol to PraCMan after completing a 36-h training course for medical assistants and installating the PraCMan software. Reimbursement for practices is given by 80€per quarter for each participating patient. Patients with CHF, T2DM or COPD participating in the HZV program may enrol to PraCMan if their predicted likelihood for hospital admission (LOH) is within the upper quartile of health plan patients [12,15]. The LOH is routinely assessed by the AOK using a validated prediction algorithm for analysis of demographic data, ICD-10 diagnoses, healthcare costs and hospital admissions within the preceding 18 months [16,17].

The PraCMan intervention is provided by medical assistants under GP-guidance and comprises three major elements [12]:

1. Assessment is performed by medical assistants using a standardised software protocol to record a comprehensive medical status. The GP and medical assistant discuss the results to identify the patient’s individual care needs. Individual treatment targets are determined in discussion and agreement between patients and caregivers. Patients receive a folder comprising a list of agreed individual treatment targets, disease-specific information leaflets, symptom diaries, action plans for self-management, medical reports, laboratory results and medication lists.
2. Monitoring is provided by medical assistants via regular telephone follow-up. Standardised monitoring items such as symptom scores and laboratory analyses are recorded and evaluated using the PraCMan software to assess the risk for deterioration of health status and the urgency of re-evaluation. Content and interval of follow-ups are decided on an individual basis for each patient via consultation between patient, medical assistant and GP.
3. Training of caregivers is provided to ensure intervention quality. After a mandatory 36-h introduction training (20 h self-study, 16 h interactive workshop) for medical assistants, practice teams receive yearly 8-h-workshops for training of the PraCMan intervention and communication techniques according to a standardised curriculum.

### Data acquisition and outcome parameters

Secondary patient data recorded for reimbursement purposes by the AOK was pseudonymised and supplied to the Department of General Practice and Health Services Research, University Hospital Heidelberg. Data storage and extraction was performed with MySQL Community Server x64 (Oracle Corporation, Redwood Shores, CA, USA). All national and institutional guidelines concerning data acquisition for retrospective analyses were followed.

**Figure 1.**
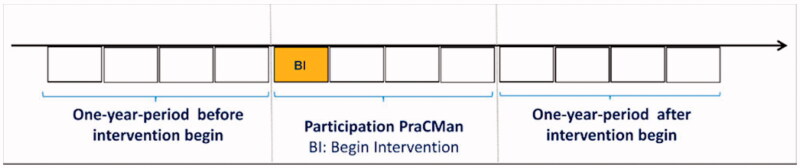
Observation periods.

The obtained data set comprised age, gender, diagnoses according to ICD-10, nursing care level and accounting data on hospital stays. The nursing care level rates the individual need for nursing care on an integral scale of one (minor impairment) to five (severe impairment) and is determined on assessment by the Medical Control Service (German: ‘Medizinischer Kontrolldienst’), an independent and governmentally supervised institution. Patients’ overall morbidity was assessed by the Charlson index, which was determined by diagnoses according to ICD-10 [18]. Number of hospital admissions and costs for hospitalisation in €were determined by the recorded Diagnosis Related Groups (DRG) codes used for reimbursement of inpatient medical services in Germany. For each patient the outcomes were determined for the one-year-periods before and 12 months after beginning of participation in the intervention. The observation periods are displayed in Figure 1.

### Statistical analysis

Propensity-score matching was performed using according to Rosenbaum and Rubin [19] using the following matching variables: age, gender, Charlson index, nursing care level, index condition and LOH. The package ‘Matching’ [20], running R Version 3.5.3 × 64, was used for the matching procedure. Controls were matched using ‘1:1 nearest-neighbour matching.’ This means that for each case individual *i* one control individual with the smallest ‘distance’ from individual *i* is selected. In our context, the distance is given by the propensity score. Both, cases and controls were recruited from the PraCMan proposal list (German: Vorschlagsliste). This list is based on an algorithm to determine high risk for hospitalisation according to LOH (please see ‘Intervention’ section). For evaluation of the matching results, the ‘MatchBalance’ function of this package was applied. This function cheques the quality of the matching for each matching variable. Depending on the scaling of the particular variable, proportions, means, and distributions were checked. We additionally calculated standardised differences for each matching variable to assess the balance between cases and matched controls. Cases and controls with death during the observation time were not included in the analysis. To calculate frequencies, rates and percentages, we used SAS PROC SQL (SAS V.9.4 × 64, SAS Institute). The R’s package ‘rateratio’ was used to calculate the risk ratios for the event ‘hospitalisation.’ The method ‘rateratio.test’ performs exact rate ratio tests based on Poisson counts and additionally calculates *p*-values and confidence intervals. For univariable nonparametric comparisons of matched cases and controls, we used R’s function ‘wilcox.test’ (Wilcoxon test). Pearson’s chi-squared test was used to compare frequencies. For all analyses, results were considered statistically significant if the *p* value was 0.05 or less.

## Results

In total, 6148 patients participated in the PraCMan intervention during the observation period. According to propensity score matching, 6148 matched controls were selected from a pool of 63,446 patients not participating in the PraCMan intervention. Table 1 shows the patient characteristics of intervention and matched control group. There were no differences between cases and matched controls for any patient characteristic.

**Table 1.** Patient characteristics.

	PraCMan	Matched controls	Standardised difference
Number of patients	6148	6148	
Male^a^ (*n*; %)	3394 (55.2%)	3443 (56.0%)	0.016
Age^a^ (mean ± SD)	75.6 ± 9.8	75.5 ± 9.8	0.009
Need for nursing care^a^ (*n*; %)	910 (14.8%)	900 (14.6%)	0.007
Charlson index^a^			0.018
Mean; 95%-CI	4.57; [4.51, 4.64]	4.53; [4.47; 4.59]	
Median; 25% perc.; 75% perc.	4.0; 3.0; 6.0	4.0; 3.0; 6.0	
Chronic index condition			
CHF^a^ (*n*; %)	4679 (76.1%)	4666 (75.9%)	0.003
T2DM^a^ (*n*; %)	3277 (53.3%)	3240 (52.7%)	0.012
COPD^a^ (*n*; %)	2803 (45.6%)	2779 (45.2%)	0.008

^a^Difference of percentage or mean: not significant.

CHF: chronic heart failure; COPD: chronic obstructive pulmonary disease; T2DM: type 2 diabetes mellitus.

In the one-year period before intervention began the per-patient hospitalisation rate was 3.5% higher for patients in the intervention group compared to control (*p* < 0.05). In the one-year period 12 months after beginning of the intervention the per-patient hospitalisation rate was 8.3% lower in the intervention group than control (*p* < 0.001). Per-patient hospitalisation costs in the pre-interventional period were 3.1% higher for patients in the intervention group compared to control (*p:* n.s.). In the observation period after beginning of the intervention per-patient hospitalisation costs were 9.4% lower in favour of the intervention group (*p* < 0.001). Average per-patient hospitalisation rates and costs for the observation periods are shown in Tables 2 and 3. The ‘Difference in Differences’ shows the difference between cases and controls regarding their particular change from the year before intervention to the year after intervention. In 2017, we had an overall average hospitalisation rate of 0.287 and average hospitalisation costs of 1243€per patient participating in the specific primary care-centred programme (‘Hausarztzentrierte Versorgung,’ HZV) for *N* = 1,037,093 patients. No significant difference was found with regard to the mortality rate at the end of the one-year observation period (cases: 11.87%, controls: 11.88%, *p:* n.s.).

**Table 2.** Average patient hospitalisation rates.

	Year before intervention	Year after intervention
PraCMan		
Mean [95%-CI]	1.112 [1.078; 1.146]	0.787 [0.754; 0.820]
Median; 25% Perc.; 75% Perc.	1.0; 0.0; 2.0	0.0; 0.0; 1.0
Matched Controls		
Mean [95%-CI]	1.074 [1.041; 1.107]	0.858 [0.824; 0.892]
Median; 25% Perc.; 75% Perc.	1.0; 0.0; 2.0	0.0; 0.0; 1.0
Risk Ratio		
(PraCMan vs. Controls)		
RR [95%-CI]	1.035 [1.001; 1.071]	0.917 [0.882; 0.954]
	*p* < 0.05	*p* < 0.001
Reduction of hospitalisations		
(DID: ΔPraCMan–ΔControls)		
Mean; 95%-CI	−0.1090; [−0.1688; −0.0491]	
Median; 25% Perc.; 75% Perc.	0.0; −1.0; 1.0	
	*p* < 0.001	

CI: confidence interval; DID: differences-in-differences; Perc.: percentile.

**Table 3.** Average patient hospitalisation costs in €.

	Year before intervention	Year after intervention
PraCMan		
Mean [95%-CI]	4570 [4348; 4792]	3315 [3114; 3516]
Median; 25% Perc.; 75% Perc.	2031.46; 0.0; 5794.03	0.0; 0.00; 3371.46
Matched Controls		
Mean [95%-CI]	4431 [4229; 4633]	3659 [3459; 3859]
Median; 25% Perc.; 75% Perc.	1811.03; 0.00; 5592.84	0.00; 0.00; 4133.72
Reduction of costs		
(DID: ΔPraCMan–ΔControls)		
Mean; 95%-CI	−482.66 [−865.02, −100.30])	
Median; 25% Perc.; 75% Perc.	−5700.04; 0.00; 4884.19	
	*p* < 0.001	

CI: confidence interval; DID: differences-in-differences; Perc.: percentile.

## Discussion

### Main findings

This is the first study to assess the effectiveness of a large-scale primary care disease management intervention for chronically ill patients in Germany using high-volume reimbursement data. The analysis of secondary data from 12,296 patients showed that participation in the PraCMan programme is associated with a lower rate of all-cause hospitalisations and lower hospitalisation costs compared to matched controls at the end of the one-year observation period.

### Interpretation of results

The effectiveness of disease management in reducing all-cause hospitalisations has already been shown in previous research, most notably in patients with CHF [11,22,23]. With regard to T2DM, there is firm evidence that disease management interventions improve glycaemic control [24–26]. For COPD, study results assessing efficacy of disease management are conflicting [9,27]. The results of this analysis suggest that the PracMan programme may be an effective approach to prevent avoidable hospitalisations. Avoiding unnecessary hospitalisation may help to reduce the large burden of medical treatment, particularly for patients with multiple chronic conditions. Reduction of hospitalisations and costs was also observed in the control group. This can be explained by the regression towards the mean phenomenon since due to matching, controls had a high rate of hospitalisations in the first year before intervention, which naturally decreased at the end of the observation period. In contrast to known disease management approaches focussing on single interventions for a specific chronic condition, PraCMan unites multiple interventions to a comprehensive care concept addressing CHF, T2DM and COPD. We postulate that three characteristics contribute to its effectiveness: (1) PraCMan unites standardised patient assessment, individualised case management and monitoring as well as continuous training of caregivers to one comprehensive care concept. (2) PraCMan allows treatment of several chronic conditions by one standardised approach. (3) PraCMan was designed to strengthen primary care, which may be the most effective mainstay for treating patients with chronic conditions, since individual medical and non-medical patient needs are best known by treating GPs and their teams.

Avoidable hospitalisation will be a driving cost factor for health care systems, which will be challenged by the rising prevalence of chronic diseases in the future [2,21]. Until today, disease management inventions have not been proven to be cost-effective. Findings of this analysis indicate an average reduction of hospitalisation costs per year of 483€per patient participating in PraCMan. Whether the administrative effort of practices is compensated by financial reimbursement of 80€per quarter and patient may not be answered by this analysis. However, long-term participation of GP practices may be seen as a potential indicator of adequate compensation. At the same time, the patient-sided benefits of avoiding hospitalisation and improved quality of life, as shown by the PraCMan trial, need to be emphasised [13].

### Strength and limitations

To our knowledge, this is the first study indicating that a comprehensive disease management programme for multiple chronic conditions in routine primary care has a beneficial effect on the rate of hospital admissions and hospitalisation costs. We conducted a reimbursement data analysis due to its high number of cases and statistical power, allowing inclusion of secondary data from 12,296 patients. Nevertheless, limitations to this study are given by its retrospective design and the nature of reimbursement data. Even if our matching was excellent for the observed covariables, we could not control for potentially relevant unobserved confounders, such as socioeconomic variables, which were not available. Furthermore, taking part in the PraCMan intervention is an ‘add on’ to the HZV programme; therefore, it is possible that some matched controls actively refused to additionally take part in PraCMan, which may be a potential source of selection bias. Due to eligibility criteria, patients are at high risk for hospitalisation at the beginning of the intervention and the effect of avoiding hospitalisation is presumably the highest within the first year. No conclusion can be drawn regarding long-term effects of PraCMan since the provided data limited the follow-up to one year. It is unclear if the intervention may also be suitable for patients with less severe chronic index conditions. Future prospective studies may address these aspects.

## Conclusion

This large cohort study in a primary care setting showed that the PraCMan intervention may be associated with a lower rate of all-cause hospital admissions and hospitalisation costs compared to usual care. Further studies may assess long-term effects of PraCMan and its efficacy in preventing known complications of chronic diseases. Large-scale implementation of PraCMan in primary care settings may be considered for chronically ill patients after its adequate evaluation in future RCTs.

## References

[1] Irving G, Neves AL, Dambha-Miller H, et al. International variations in primary care physician consultation time: a systematic review of 67 countries. BMJ Open. 2017;7(10):e017902. 10.1136/bmjopen-2017-017902 PMC5695512 29118053

[2] Wolff JL, Starfield B, Anderson G. Prevalence, expenditures, and complications of multiple chronic conditions in the elderly. Arch Intern Med. 2002;162(20):2269–2276. 12418941 10.1001/archinte.162.20.2269

[3] Ghorob A, Bodenheimer T. Sharing the care to improve access to primary care. N Engl J Med. 2012;366(21):1955–1957. 22621625 10.1056/NEJMp1202775

[4] Mergenthal K, Beyer M, Gerlach FM, et al. Sharing responsibilities within the general practice team – a cross-sectional study of task delegation in Germany. PLoS One. 2016;11(6):e0157248. 27280415 10.1371/journal.pone.0157248 PMC4900540

[5] Freund T, Everett C, Griffiths P, et al. Skill mix, roles and remuneration in the primary care workforce: who are the healthcare professionals in the primary care teams across the world? Int J Nurs Stud. 2015;52(3):727–743. 25577306 10.1016/j.ijnurstu.2014.11.014

[6] Senft JD, Wensing M, Poss-Doering R, et al. Effect of involving certified healthcare assistants in primary care in Germany: a cross-sectional study. BMJ Open. 2019;9(12):e033325. 10.1136/bmjopen-2019-033325 PMC6936982 31888935

[7] Doherty M, Jenkins W, Richardson H, et al. Efficacy and cost-effectiveness of nurse-led care involving education and engagement of patients and a treat-to-target urate-lowering strategy versus usual care for gout: a randomised controlled trial. Lancet. 2018;392(10156):1403–1412. 30343856 10.1016/S0140-6736(18)32158-5 PMC6196879

[8] Healey EL, Main CJ, Ryan S, et al. A nurse-led clinic for patients consulting with osteoarthritis in general practice: development and impact of training in a cluster randomised controlled trial. BMC Fam Pract. 2016;17(1):173. 28003026 10.1186/s12875-016-0568-y PMC5178095

[9] Kruis AL, Smidt N, Assendelft WJJ, et al. Integrated disease management interventions for patients with chronic obstructive pulmonary disease. Cochrane Database Syst Rev. 2013;10:CD009437. 10.1002/14651858.CD009437.pub2 24108523

[10] Rodriguez HP, Friedberg MW, Vargas-Bustamante A, et al. The impact of integrating medical assistants and community health workers on diabetes care management in community health centers. BMC Health Serv Res. 2018;18:875. 30458778 10.1186/s12913-018-3710-9 PMC6247511

[11] Takeda A, Martin N, Taylor RS, et al. Disease management interventions for heart failure. Cochrane Database Syst Rev. 2019;1:CD002752. 30620776 10.1002/14651858.CD002752.pub4 PMC6492456

[12] Freund T, Wensing M, Mahler C, et al. Development of a primary care-based complex care management intervention for chronically ill patients at high risk for hospitalization: a study protocol. Implement Sci. 2010;5:70. 20858242 10.1186/1748-5908-5-70 PMC2949784

[13] Freund T, Peters-Klimm F, Boyd CM, et al. Medical assistant-based care management for high-risk patients in small primary care practices: a cluster randomized clinical trial. Ann Intern Med. 2016;164(5):323–330. 26833209 10.7326/M14-2403

[14] Laux G, Kaufmann-Kolle P, Bauer E, et al. Evaluation of family doctor centred medical care based on AOK routine data in Baden-Württemberg. Z Evid Fortbild Qual Gesundhwes. 2013;107(6):372–378. 24075678 10.1016/j.zefq.2013.07.001

[15] Freund T, Peters-Klimm F, Rochon J, et al. Primary care practice-based care management for chronically ill patients (PraCMan): study protocol for a cluster randomized controlled trial [ISRCTN56104508]. Trials. 2011;12:163. 21714883 10.1186/1745-6215-12-163 PMC3141533

[16] Haupt C, Guenster C. Statistical risk models: applicability for the optimization of patient care. Monitor Versorgungsforschung. 2013;1:36–39.

[17] Freund T, Gondan M, Rochon J, et al. Comparison of physician referral and insurance claims data-based risk prediction as approaches to identify patients for care management in primary care: an observational study. BMC Fam Pract. 2013;14:157. 24138411 10.1186/1471-2296-14-157 PMC3856595

[18] Sundararajan V, Henderson T, Perry C, ET AL. New ICD-10 version of the charlson comorbidity index predicted in-hospital mortality. J Clin Epidemiol. 2004;57(12):1288–1294. 15617955 10.1016/j.jclinepi.2004.03.012

[19] Rosenbaum PR, Rubin DB. The central role of the propensity score in observational studies for causal effects. Biometrika. 1983;70(1):41–55.

[20] Sekhon JS. Multivariate and propensity score matching software with automated balance optimization: the matching package for *R*. J Stat Soft. 2011;42(7):1–52. Available from: http://www.jstatsoft.org/v42/i07/

[21] Starfield B, Lemke KW, Bernhardt T, ET AL. Comorbidity: implications for the importance of primary care in “'case' management”. Ann Fam Med. 2003;1(1):8–14. 15043174 10.1370/afm.1 PMC1466556

[22] Whellan DJ, Hasselblad V, Peterson E, ET AL. Metaanalysis and review of heart failure disease management randomized controlled clinical trials. Am Heart J. 2005;149(4):722–729. 15990759 10.1016/j.ahj.2004.09.023

[23] McAlister FA, Lawson FM, Teo KK, et al. A systematic review of randomized trials of disease management programs in heart failure. Am J Med. 2001;110(5):378–384. 11286953 10.1016/s0002-9343(00)00743-9

[24] Pimouguet C, Le Goff M, Thiébaut R, et al. Effectiveness of disease-management programs for improving diabetes care: a meta-analysis. CMAJ. 2011;183(2):E115–127. 21149524 10.1503/cmaj.091786 PMC3033953

[25] Rothman RL, Malone R, Bryant B, et al. A randomized trial of a primary care-based disease management program to improve cardiovascular risk factors and glycated hemoglobin levels in patients with diabetes. Am J Med. 2005;118(3):276–284. 15745726 10.1016/j.amjmed.2004.09.017

[26] Tricco AC, Ivers NM, Grimshaw JM, et al. Effectiveness of quality improvement strategies on the management of diabetes: a systematic review and meta-analysis. Lancet. 2012;379(9833):2252–2261. 22683130 10.1016/S0140-6736(12)60480-2

[27] Kruis AL, Boland MRS, Assendelft WJJ, et al. Effectiveness of integrated disease management for primary care chronic obstructive pulmonary disease patients: results of cluster randomised trial. Br Med J. 2014;349(11):g5392. 25209620 10.1136/bmj.g5392 PMC4160285

